# Early Cognitive Outcomes After Carotid Endarterectomy in Asymptomatic Stenosis: A Pilot Study

**DOI:** 10.7759/cureus.96559

**Published:** 2025-11-11

**Authors:** Ladislav Kocan, Rudolf Sudzina, Hana Kocanová, Martina Závacká, Jana Pobehová, Dusan Rybár, Martina Dudová, Miroslav Ferencík, Janka Vašková

**Affiliations:** 1 Department of Anaesthesiology and Intensive Medicine, East Slovak Institute of Cardiovascular Diseases, Košice, SVK; 2 Department of Anaesthesiology and Intensive Medicine, Railway Hospital, Košice, SVK; 3 Department of Vascular Surgery, East Slovak Institute of Cardiovascular Diseases, Košice, SVK; 4 Department of Anaesthesiology, X-Pain Clinic, Bojnice, SVK; 5 Department of Medical Biology, Pavol Jozef Šafárik University in Košice's Faculty of Medicine, Košice, SVK

**Keywords:** carotid endarterectomy, cerebral oximetry, cognition, neurological status, postoperative cognitive dysfunction

## Abstract

Background

Perioperative neurocognitive disorders (PNDs) are important determinants of postoperative quality of life. Evidence on early cognitive outcomes after carotid endarterectomy (CEA) in neurologically intact, asymptomatic patients remains limited, and reported incidences vary widely.

Methodology

This single-center prospective observational pilot study (ClinicalTrials.gov NCT06391866) enrolled 18 statin-treated adults (mean age 78.3 ± 4.9 years) with asymptomatic ≥70% ICA stenosis undergoing CEA under balanced general anesthesia with bilateral near-infrared spectroscopy (NIRS) and entropy monitoring. Neurological (National Institutes of Health Stroke Scale (NIHSS)) and Montreal Cognitive Assessment (MoCA) tests were done preoperatively and 24 hours postoperatively; patients with delirium were excluded. Subjective cognitive change was assessed at 30 days by interview. Early POCD was a ≥2-point MoCA decline.

Results

Mean clamp time was 29.1 ± 6.4 minutes; no shunts were used. NIRS showed no ≥25% decrease or >10% increase from baseline after unclamping. NIHSS was unchanged (median 1 → 1, p > 0.05). MoCA fell non-significantly from 30.0 ± 1.8 to 29.6 ± 2.3 (p > 0.05); one patient (5.6%) met criteria for early POCD. At 30 days, one patient reported improvement, 13 had no change, three were lost to follow-up, and one died of non-neurological causes. No strokes, transient ischemic attacks (TIAs), or hyperperfusion occurred.

Conclusions

In this small pilot study of neurologically intact, asymptomatic patients undergoing CEA with meticulous monitoring, early postoperative cognitive decline was rare, neurological function remained stable, and no major perioperative complications occurred. These findings suggest that with careful intraoperative management, CEA is generally safe regarding early cognitive outcomes in this population.

## Introduction

Perioperative neurocognitive disorders (PNDs), encompassing postoperative delirium and postoperative cognitive dysfunction (POCD), are recognized as important determinants of recovery, independence, and quality of life after surgery. Their etiology is multifactorial: patient-specific vulnerabilities, such as advanced age, vascular risk factors, and metabolic comorbidities, interact with surgical magnitude and anesthetic strategy to influence risk.

In the context of carotid endarterectomy (CEA), the literature reports a wide range of POCD incidences, often between 15% and 30% in mixed symptomatic and asymptomatic cohorts. A recent meta-analysis of 3,459 CEAs identified cerebral hyperperfusion, high-grade internal carotid artery (ICA) stenosis, and diabetes mellitus as consistent perioperative predictors, whereas statin therapy appeared protective [1]. These observations highlight the interplay between systemic atherosclerosis, cerebral hemodynamics, and neuroinflammatory processes [2].

Prospective studies suggest that perioperative hemodynamic disturbances, especially intraoperative cerebral desaturation and postoperative hyperperfusion, may contribute to neurocognitive decline [3]. Near-infrared spectroscopy (NIRS) has revealed a dose-response relationship between the depth of intraoperative desaturation and later cognitive impairment, with sex and diabetes as amplifying factors. Conversely, modest intraoperative increases in cerebral saturation may signal hyperperfusion, which has been linked to sustained cognitive deficits up to two years post-CEA [4].

Despite the stroke-preventive benefit of CEA in selected asymptomatic patients, there is a need to clarify whether the procedure carries a measurable early cognitive penalty in this lower-risk group. Many published studies have methodological limitations, including heterogeneous populations, inconsistent neuropsychological testing schedules, and the absence of standardized brain monitoring. Furthermore, there is a paucity of prospective data specifically targeting neurologically intact patients with asymptomatic high-grade ICA stenosis under contemporary multimodal anesthetic and surgical care [5-7].

This single-center prospective observational pilot study was designed to explore the feasibility of integrating detailed perioperative hemodynamic and cerebral oxygenation monitoring with standardized cognitive testing before and after CEA in asymptomatic patients. The aims were to describe early postoperative changes in Montreal Cognitive Assessment (MoCA) scores, assess whether intraoperative regional cerebral oxygen saturation (rSO₂) decreases or increases relate to cognitive outcome, and provide preliminary data to inform the design and sample size calculation of a future multicenter trial powered to detect clinically relevant differences in POCD incidence.

## Materials and methods

Study design and ethical approval

This was a single-center, prospective observational pilot study conducted at the East Slovak Institute for Cardiovascular Diseases (Košice, Slovakia) between April 2024 and November 2025. The study was designed to assess the feasibility of combined perioperative hemodynamic and cerebral oximetry monitoring with standardized cognitive testing before and after CEA in asymptomatic patients. The protocol was approved by the institutional ethics committee (No. A2012024/VUSCH/EK), registered at ClinicalTrials.gov (NCT06391866), and performed in accordance with the Declaration of Helsinki and Good Clinical Practice. All participants provided written informed consent.

Sample size rationale

As a pilot study, the sample size was determined pragmatically based on the number of eligible patients within the study period, surgical scheduling, and research personnel availability. No formal a priori power calculation was performed. The data obtained were intended to inform a future multicenter study with adequate statistical power.

Based on the observed incidence of early POCD in this cohort (5.6%), a sample size calculation indicates that approximately 78 patients per group would be required to detect a difference from a hypothetical 20% incidence with 80% power and α = 0.05.

Participants

Adults aged 18-80 years with duplex- or CT-confirmed ≥70% ICA stenosis, neurologically intact, and scheduled for primary CEA under general anesthesia were screened. All had received high-intensity statin therapy (atorvastatin ≥40 mg/day or rosuvastatin ≥20 mg/day) for at least one year prior to surgery.

Exclusion criteria included the following: previous ipsilateral CEA or carotid stenting; stroke with modified Rankin score >3 within the preceding six months; diagnosed dementia, major psychiatric illness, or severe sensory impairment precluding cognitive testing; conditions likely to compromise protocol adherence or outcome assessment; and refusal to participate.

Preoperative assessment

A board-certified neurologist examined all candidates the day before surgery using the National Institutes of Health Stroke Scale (NIHSS) [8]. Global cognition was assessed with the Slovak-validated MoCA [9]. Only patients without clinical signs of delirium or significant neurological deficit were enrolled.

Surgical technique

Two senior vascular surgeons performed all CEAs. Patients were positioned in the semi-sitting (“beach-chair”) position with mild neck extension and contralateral rotation. Standard exposure and dissection of the carotid bifurcation were performed. After systemic heparinization (10,000 IU), vessels were clamped in the sequence common carotid artery → ICA → external carotid artery → superior thyroid artery. The default technique was longitudinal endarterectomy with Dacron patch closure; eversion endarterectomy was performed when anatomy was favorable.

Bilateral rSO₂ was monitored using NIRS (INVOS™, Medtronic, Mansfield, MA). A >25% fall in ipsilateral rSO₂ from baseline prompted intraluminal shunt placement. For the purposes of this study, we also recorded any increase >10% in rSO₂ from baseline after unclamping as a potential surrogate marker of hyperperfusion.

Anesthetic management and intraoperative monitoring

General anesthesia was induced with propofol (2-2.5 mg/kg) and fentanyl or sufentanil, with midazolam as required. Neuromuscular blockade was achieved with atracurium or rocuronium. Maintenance used low-flow sevoflurane (≤1 minimum alveolar concentration (MAC)) in oxygen-air with opioid supplementation as needed. Depth of hypnosis was titrated with entropy monitoring (GE S/5 Entropy™, GE Healthcare, Chicago, IL): State Entropy (SE) was maintained between 40 and 60; a Response Entropy (RE)-SE gap >10 prompted additional analgesia or an anesthetic.

Ventilation was adjusted to maintain normocapnia (PaCO₂ 4.5-5.5 kPa). Standard monitoring included five-lead ECG, pulse oximetry, capnography, a radial arterial catheter for continuous blood pressure, bilateral NIRS probes positioned 2 cm above the supraorbital ridge, and esophageal temperature monitoring. Mean arterial pressure (MAP) was maintained within ±20% of baseline before clamping and ≥80 mmHg during clamping.

Postoperative neurological and cognitive assessment

NIHSS and MoCA were repeated 24 hours postoperatively by the same neurologist. To minimize confounding by delirium, all patients were screened clinically prior to testing; those with features of delirium were excluded from cognitive assessment. The 24-hour time point was chosen to capture early POCD as defined in previous carotid surgery studies, with the acknowledgment that it may overlap with the typical window for postoperative delirium; this is discussed as a limitation.

Follow-up

At 30 days, patients were contacted for a structured exploratory interview assessing perceived changes in memory, concentration, reading, and writing using a visual analogue scale (0% = markedly worse, 100% = markedly improved). This was considered a supplementary, patient-centered measure and not a primary cognitive endpoint.

Data collection and outcomes

Anesthesia records provided clamp duration, nadir, and peak ipsilateral rSO₂, nadir SE, peak RE-SE gap, and frequency/duration of MAP excursions >25% from baseline.

Primary outcomes include change in MoCA score and incidence of early POCD (≥2-point MoCA decline). Secondary outcomes include perioperative neurological events, need for shunt, other complications, and self-reported cognitive status at 30 days.

Statistical analysis

Data were captured in REDCap (Informatics Team, Vanderbilt University, Nashville, TN) and analyzed using IBM SPSS Statistics version 26 (IBM Corp., Armonk, NY). Normality was assessed with the Shapiro-Wilk. Continuous variables are reported as mean ± SD or median (interquartile range (IQR)) and compared with paired t-tests or Wilcoxon signed-rank tests. Categorical variables are expressed as counts (percentages) and compared with Fisher’s exact test. Associations between hemodynamic parameters (including rSO₂ increases and decreases) and MoCA change were explored with Spearman’s rho. A two-sided p < 0.05 was considered statistically significant.

## Results

Patient characteristics

Eighteen patients (10 men, eight women; age range 69-87 years, mean age 78.3) were included in the study. Comorbid conditions were common. Cardiac disorders included ischemic heart disease (16/18), cardiac arrhythmia (8/18), and chronic heart failure (2/18). Peripheral and large-vessel pathology was represented by peripheral arterial disease (10/18) and an asymptomatic aortic aneurysm (2/18). Respiratory disorders included chronic obstructive pulmonary disease (1/18), bronchial asthma (1/18), previous pulmonary embolism (1/18), and post-COVID condition (2/18). Chronic renal insufficiency was present in 3 patients. Neurological history included previous transient ischemic attack (5/18) and dementia (1/18), with no cases of Parkinsonism. Among metabolic disorders, diabetes mellitus was present in four patients, and hyperlipidemia in all (18/18). Thyroid disease was reported in two out of 10 patients, and one patient had a history of malignancy. No patient required intraoperative shunt placement. The surgical procedure was evenly distributed, with nine patients undergoing left-sided and nine right-sided carotid surgery.

Anesthetic and surgical data

Premedication included oxazepam in nine patients (mean dose 2.81 mg) and melatonin in three (mean dose 0.63 mg). Intravenous anesthetic agents included propofol (mean dose 105.3 mg), midazolam (1 mg), and sufentanil (6.6 mL). Mean duration of anesthesia was 86 minutes; mean carotid clamp time was 29.1 ± 6.4 minutes. Postoperative analgesia comprised metamizole (0.84 g) and paracetamol (0.75 g) (Table 1).

**Table 1:** Premedication and intra-operative intravenous anesthetic consumption, with statistical summary of total anesthesia duration and carotid clamp time. Not applicable (N/A)

Premedication	Administered/not administered (n)	Min dose	Max dose	Mean dose
Oxazepam (mg)	9.9	0	5	2.81
Melatonin (mg)	3/15	0	5	0.63
Anesthesia (IV anesthetics)				
Propofol (mg)	18/0	50	200	105.3
Midazolam (mg)	7/11	0	5	1
Sufentanil (mL)	18/0	4	10	6.6
Anesthesia duration (minutes)	N/A	69	120	86
Clamp time (minutes)	N/A	15	60	29.1
Post-operative analgesia				
Metamizole (g)	9.7	0	2.5	0.84
Paracetamol (g)	14.4	0	1	0.75

Hemodynamic parameters

**Figure 1:**
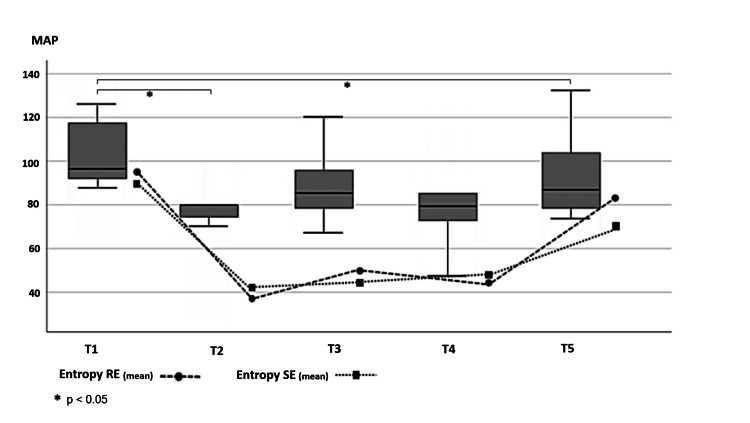
Box-and-whisker plots showing mean arterial pressure at the five perioperative time points (T1-T5) alongside a line graph illustrating the mean Response Entropy (RE) and State Entropy (SE) values recorded over the same interval.

Median baseline MAP at T1 (before induction) was 95 mmHg, decreasing to 85 mmHg before clamping (T2) and remaining stable during clamping (T3) and emergence (T5). After unclamping (T4), MAP decreased further to 80 mmHg. Compared with baseline, this decline was statistically significant (p < 0.05) but remained within target ranges (Figure 1).

Cerebral oximetry and entropy parameters

NIRS revealed no ≥25% decrease in rSO₂ from baseline at any intraoperative stage, and thus no patient met the threshold for shunt insertion.

Evaluation of rSO₂ increases showed that after unclamping, ipsilateral rSO₂ increased by a mean of +4.3% (range +1% to +8%) relative to the pre-clamp baseline. No patient exhibited an increase >10%, which was our predefined surrogate for hyperperfusion. Contralateral rSO₂ changes remained within ±5% of baseline throughout (Table 2).

**Table 2:** Comparison of NIRS values between the operated and intact side across different perioperative time points. Time points of measurement: T1 - before induction of general anesthesia; T2 - before clamping of the internal carotid artery; T3 - during clamping of the internal carotid artery; T4 - after unclamping of the internal carotid artery; T5 - after emergence from general anesthesia. Statistically significant at p > 0.05. NIRS, near-infrared spectroscopy

	Min	Max	Median	SD	95% CI		p-value
					Lower	Upper	
NIRS operated side (T1)	48	70	63.5	7.6	-5.59	10.26	>0.05
NIRS intact side (T1)	48	72	64.5				
NIRS operated side (T2)	32	68	62.5	12.2	-7.30	10.64	>0.05
NIRS intact side (T2)	42	82	66				
NIRS operated side (T3)	40	84	55.5	12.9	-7.22	14.87	>0.05
NIRS intact side (T3)	41	71	63				
NIRS operated side (T4)	53	85	62	7.9	-11.41	7.12	>0.05
NIRS intact side (T4)	54	68	61.5				
NIRS operated side (T5)	54	77	63	11.6	-6.35	12.15	>0.05
NIRS intact side (T5)	56	78	67.2				

SE and RE decreased significantly from baseline during maintenance phases (T2-T4) and rose predictably during emergence (T5) (all p < 0.05), consistent with intended anesthetic depth. The RE-SE gap remained <10 in most cases, with occasional transient increases promptly addressed with additional analgesia or an anesthetic (Figure 1).

Neurological and cognitive outcomes

MoCA scores decreased non-significantly from 30.0 ± 1.8 preoperatively to 29.6 ± 2.3 at 24 hours postoperatively (p > 0.05). Only one patient (5.6%) met the definition of early POCD (≥2-point decline) [6]. No patient exhibited clinical delirium at the time of testing (Table 3).

**Table 3:** Comparison of MoCA and overall neurological status (NIHSS) before and 24 hours after the procedure. Statistically significant at p > 0.05. MoCA, Montreal Cognitive Assessment; NIHSS, National Institutes of Health Stroke Scale

	Min	Max	Median	SD	95% CI		p-value
					Lower	Upper	
NIHSS before the procedure	0	2	1	0.7	-0.84	0.34	>0.05
NIHSS 24 hours after the procedure	0	4	1				
MoCA before the procedure	26	30	29.3	0.7	-0.93	1.79	>0.05
MoCA 24 hours after the procedure	26	30	29.1				

Follow-up at 30 days

One patient (10% of those reached) reported subjective cognitive improvement, 13 (72%) reported no change, three were unreachable, and one had died from non-neurological causes. No strokes, transient ischemic attacks, or hyperperfusion syndromes were documented during the follow-up period.

## Discussion

CEA is carried out mainly to prevent stroke, yet its effect on higher brain functions remains debated. Meta-analyses and narrative reviews reveal considerable variability; some studies describe postoperative cognitive improvement, others a transient decline, and still others no measurable change at all [10]. Such heterogeneity is largely attributable to methodological differences, including the mixing of symptomatic and asymptomatic cohorts, variability in neuropsychological test batteries, and inconsistent follow-up schedules [11]. 

Our prospective pilot study was designed to reduce some of these sources of variability by enrolling only neurologically intact individuals with asymptomatic, high-grade ICA stenosis, all of whom were receiving moderate- to high-intensity statin therapy. CEA was performed under balanced general anaesthesia guided by NIRS and EEG entropy monitoring. We observed no significant decrease in MoCA scores 24 hours after surgery and no self-reported cognitive decline at 30 days [4]. These results differ from the 15-30% early cognitive deterioration rates reported in broader mixed cohorts and agree with recent tightly phenotyped studies demonstrating stable cognition when meticulous monitoring is applied [12].

Two mechanisms may explain the favorable cognitive profile observed. First, postoperative cerebral hyperperfusion is a recognized driver of sustained cognitive decline up to two years after CEA [3]. In our study, no patient reached clinical or NIRS criteria for hyperperfusion, and increases in rSO₂ after unclamping never exceeded 10% from baseline, our predefined surrogate threshold for hyperperfusion risk. Second, previous research has demonstrated a relationship between the depth of intraoperative cerebral desaturation and later neuropsychological performance [4]. In our series, regional oxygen saturation never fell by 25% or more from baseline, so shunt placement was unnecessary; this stability likely contributed to the absence of early cognitive decline despite systemic blood pressure fluctuations.

Long-term statin use also deserves emphasis. Statin therapy has been repeatedly associated with a reduced incidence of POCD after CEA [13,14].

In our cohort, universal statin use may have conferred anti-inflammatory and endothelial-stabilizing effects that dampened the neuroinflammatory cascade prompted by surgical trauma.

NIRS proved to be a practical tool for detecting intraoperative cerebral hypoperfusion. Persistently low rSO₂ values despite shunting, combined with a new focal neurological deficit, should prompt postoperative neuroimaging to rule out infarction [14,15]. In this series, no patient required a shunt, and no postoperative deficits occurred, supporting the reliability of our NIRS-guided thresholds. Critics highlight the frontal bias of NIRS and the possibility of missing subcortical ischemia [10,16]. Nevertheless, when interpreted alongside hemodynamic data, NIRS served as an effective early-warning surrogate. Future research should refine multimodal trigger points combining NIRS, transcranial Doppler velocities, and perfusion imaging to guide shunt decisions and blood pressure targets [11,17].

Another factor that warrants consideration is rigorous postoperative pain control [18,19]. Although persistent pain after CEA is uncommon, sustained nociceptive input from cervical soft-tissue trauma or cranial nerve irritation can activate microglia, promote maladaptive synaptic plasticity, and lead to central sensitization [20]. Chronic pain is increasingly associated with impairments in attention, memory, and executive function, potentially contributing to POCD [21,22].

This study has several important limitations. First, the small sample size (n = 18) limits statistical power for detecting independent predictors such as clamp time or anesthetic dose. As a pilot study, our primary aim was to assess feasibility and generate preliminary data; based on the observed POCD incidence of 5.6%, approximately 78 patients per group would be needed in a future trial to detect a clinically significant difference from a 20% incidence with 80% power at α = 0.05. Second, cognitive testing at 24 hours may be influenced by transient factors, including residual anesthetic effects, analgesia, fatigue, or subclinical delirium. Although all patients were screened for delirium before testing, this early time frame may underestimate cognitive performance. Longer-term domain-specific assessments, such as the MoCA, Trail Making Test, or verbal learning tasks, are planned for future studies. Third, the 30-day structured telephone interview was an exploratory, patient-centered measure, not a primary endpoint, and lacks the objectivity of formal neuropsychological testing. Fourth, while no patient met intraoperative NIRS or clinical criteria for hyperperfusion, the absence of postoperative perfusion imaging (transcranial Doppler (TCD), single-photon emission computed tomography (SPECT), MRI) means that subclinical hyperperfusion cannot be excluded.

Within these constraints, our results suggest that carefully selected asymptomatic patients undergoing CEA under balanced general anesthesia with vigilant cerebral oximetry and statin therapy do not experience a high rate of early cognitive decline; indeed, their cognitive trajectory appears stable and may improve relative to historical mixed cohorts. These observations highlight the importance of thorough preoperative cognitive screening, consistent postoperative follow-up with sensitive instruments, intraoperative management to maintain stable cerebral oxygenation, and the continued use of vascular-protective medications such as statins. To confirm the generalizability of these findings, future adequately powered multicenter trials should incorporate multimodal monitoring, inflammatory biomarkers, postoperative perfusion imaging, and comprehensive neuropsychological batteries to establish broadly applicable brain-protective protocols for carotid surgery.

## Conclusions

In this single-center pilot feasibility study of neurologically intact, statin-treated patients with asymptomatic high-grade ICA stenosis, CEA performed under vigilant cerebral oximetry and balanced general anesthesia was associated with a low incidence of early POCD. Neither significant intraoperative rSO₂ decreases nor increases suggestive of hyperperfusion were observed.

These findings should be interpreted with caution due to the small sample size, early timing of postoperative cognitive testing, and absence of postoperative perfusion imaging. Nevertheless, they support the feasibility of combining standardized cognitive assessment with multimodal intraoperative monitoring in this patient group. Future multicenter trials with larger cohorts (estimated ≥78 patients per group), extended follow-up, and more sensitive cognitive testing are needed to confirm these results and refine brain-protective strategies in carotid surgery.

## References

[1] He J, Duan R, Qiu P (2023). The risk factors of postoperative cognitive dysfunction in patients undergoing carotid endarterectomy: an updated meta-analysis. J Cardiothorac Surg.

[2] Ozcaglayan O, K TI, Gur DO, Gumusel HK, Topcu B, Unal A (2019). Carotid arteries and vertebrobasilary system intracranial stenosis correlates with multi vessel coronary artery disease. Bratisl Lek Listy.

[3] Araya S, Akamatsu Y, Ono Y (2025). Impact of postoperative cerebral hyperperfusion on 2-year cognitive outcomes of patients undergoing carotid endarterectomy. J Neurosurg.

[4] Sándor ÁD, Czinege Z, Szabó A (2024). Cerebrovascular dysregulation and postoperative cognitive alterations after carotid endarterectomy. Geroscience.

[5] Yamazaki R, Akamatsu Y, Yoshida J (2024). Association between preoperative white matter hyperintensities and postoperative new ischemic lesions on magnetic resonance imaging in patients with cognitive decline after carotid endarterectomy. Neurosurg Rev.

[6] Somnuke P, Srishewachart P, Jiraphorncharas C (2024). Early postoperative neurocognitive complications in elderly patients: comparing those with and without preexisting mild cognitive impairment- a prospective study. BMC Geriatr.

[7] Ning Y, Guo J, Pan D (2024). The effects of carotid revascularization on 1-year cognitive performance in patients with carotid artery stenosis. J Endovasc Ther.

[8] Brott T, Adams HP Jr, Olinger CP (1989). Measurements of acute cerebral infarction: a clinical examination scale.. Stroke.

[9] Nasreddine ZS, Phillips NA, Bédirian V (2005). The Montreal Cognitive Assessment, MoCA: a brief screening tool for mild cognitive impairment. J Am Geriatr Soc.

[10] Krebs JR, Anderson EM, Fazzone B, Agaba P, Shah SK (2025). Asymptomatic carotid artery stenosis, cognitive function, and the impact of carotid revascularization: a narrative review. Ann Vasc Surg.

[11] Stilo F, Montelione N, Paolini P (2024). Current status of brain monitoring during carotid endarterectomy. JVS Vasc Insights.

[12] Relander K, Hietanen M, Ijäs P (2024). Long-term cognitive and neurovascular changes after carotid endarterectomy. J Neurol Sci.

[13] Relander K, Hietanen M, Nuotio K (2020). Cognitive dysfunction and mortality after carotid endarterectomy. Front Neurol.

[14] Heyer EJ, Mergeche JL, Bruce SS, Connolly ES (2013). Does cognitive dysfunction after carotid endarterectomy vary by statin type or dose?. Int J Brain Cogn Sci.

[15] Battaglini D, Pelosi P, Robba C (2022). The importance of neuromonitoring in non brain injured patients. Crit Care.

[16] Yang M, Yang Z, Yuan T, Feng W, Wang P (2019). A systemic review of functional near-infrared spectroscopy for stroke: current application and future directions. Front Neurol.

[17] Yu Y, Zhang K, Zhang L, Zong H, Meng L, Han R (2018). Cerebral near-infrared spectroscopy (NIRS) for perioperative monitoring of brain oxygenation in children and adults. Cochrane Database Syst Rev.

[18] Khaled M, Sabac D, Fuda M (2025). Postoperative pain and neurocognitive outcomes after noncardiac surgery: a systematic review and dose-response meta-analysis. Br J Anaesth.

[19] Wagnerova H, Goldenberg Z (2013). Transient ischemic attack in the vertebro-basilar circulation due to a hemodynamically significant variation - kinking of the extracranial section of the left vertebral artery. Bratisl Lek Listy.

[20] Patel M, Hasoon J, Diez Tafur R, Lo Bianco G, Abd-Elsayed A (2025). The impact of chronic pain on cognitive function. Brain Sci.

[21] Kelly K, Keohane E, Davy G (2025). The effect of chronic pain on memory: a systematic review and meta-analysis exploring the impact of nociceptive, neuropathic and nociplastic pain. Brain Cogn.

[22] Song Q, E S, Zhang Z, Liang Y (2024). Neuroplasticity in the transition from acute to chronic pain. Neurotherapeutics.

